# The multifaceted roles of mitochondria and their therapeutic transformation: a new perspective on triple-negative breast cancer treatment

**DOI:** 10.3389/fonc.2026.1837476

**Published:** 2026-06-18

**Authors:** Siyuan Yang, Lei Wang, Jianyun Nie

**Affiliations:** 1 Department of Breast Surgery, Peking University Cancer Hospital Yunnan Hospital, Yunnan Cancer Hospital, Third Affiliated Hospital, Kunming Medical University, Kunming, Yunnan, China; 2 Department of General Surgery, 926 Hospital of the Joint Service Support Force of the Chinese People’s Liberation Army, Kaiyuan, Yunnan, China

**Keywords:** triple-negative breast cancer, mitochondria, mitochondrial dynamics, mitochondrial metabolism, mitochondria-targeted therapy

## Abstract

Triple-negative breast cancer (TNBC) represents the most aggressive breast cancer subtype, lacking effective targeted therapies and exhibiting pronounced therapeutic resistance. Mitochondria have recently emerged as central regulators of TNBC pathogenesis, functioning beyond their traditional roles as cellular powerhouses. This article synthesizes current understanding of how mitochondrial metabolic reprogramming—particularly the synergistic hyperactivation of oxidative phosphorylation, fatty acid oxidation, and glutamine metabolism—drives TNBC progression, metastasis, and chemoresistance. We further examine the dichotomous roles of mitochondrial dynamics and mitophagy in shaping tumor cell fate, and explore how mitochondria orchestrate diverse programmed cell death pathways and immune modulation. Translational strategies targeting mitochondrial vulnerabilities, including small-molecule inhibitors, nanomaterial-based delivery systems, and combination regimens, are critically evaluated. Despite significant preclinical promise, challenges including tumor selectivity, metabolic plasticity, and clinical translation remain. By integrating mechanistic insights with emerging therapeutic innovations, this perspective highlights the transformative potential of mitochondria-targeted interventions for future TNBC management.

## 1 Introduction

Triple-negative breast cancer (TNBC) represents a specific subtype of breast cancer fundamentally characterized by the absence of estrogen receptor (ER), progesterone receptor (PR), and human epidermal growth factor receptor 2 (HER2) expression. While comprising roughly 10 - 20% of all breast cancer incidences (1–4), this is largely driven by its high recurrence rate, an aggressive tendency for early distant metastasis (predominantly to the visceral, liver, and brain), and intrinsic resistance to standard-of-care treatments (5–7). Given these clinical challenges, identifying novel therapeutic vulnerabilities and interventions remains an urgent imperative.

For decades, the prevailing dogma suggested that cancer cells fulfill their robust energetic demands primarily through aerobic glycolysis (the Warburg effect), accompanied by compromised mitochondrial oxidative phosphorylation (OXPHOS) (8). Yet, this classical view is undergoing a profound paradigm shift. Extensive research now reveals that mitochondria are exceptionally active in TNBC. They profoundly drive the malignant phenotype by dynamically regulating critical cellular events, including energy metabolism, reactive oxygen species (ROS) homeostasis, apoptosis, calcium signaling, mitochondrial dynamics, and mitochondrial quality control (MQC) (9–11). Thus, deciphering the multifaceted contributions of mitochondria to TNBC development and systematically assessing the translational feasibility of mitochondria-targeted therapies will pave the way for expanding the clinical armamentarium against this formidable disease.

## 2 Manuscript

### 3.1 Methods

#### 3.1.1 Literature search strategy

The literature was identified through systematic searches of the PubMed database from inception to March 2026. The following search strategy was employed: (Triple-Negative Breast Cancer) AND (Mitochondria). Additional relevant articles were identified by manual screening of reference lists from included papers and key reviews.

#### 3.1.2 Gene expression

According to bc-GenExMiner v5.2, differences in distributions of mitochondria-related genes among different subtype are analyzed (12). These four TNBC subtypes include the luminal androgen receptor (LAR), the mesenchymal stem-like (MLIA), the basal-like immune-activated (BLIA) and the basal-like immune-suppressed (BLIS) (13). To assess the significance of the difference in gene distributions in between the different groups, a Welch’s test is performed, as well as Dunnett-Tukey-Kramer’s tests when appropriate. Box and whisker are displayed, along with Welch’s (and Dunnett-Tukey-Kramer’s) tests for every possible population splitting criteria for a unique gene.

### 3.2 Multifaceted mitochondrial mechanisms driving TNBC malignancy

**Figure 1:**
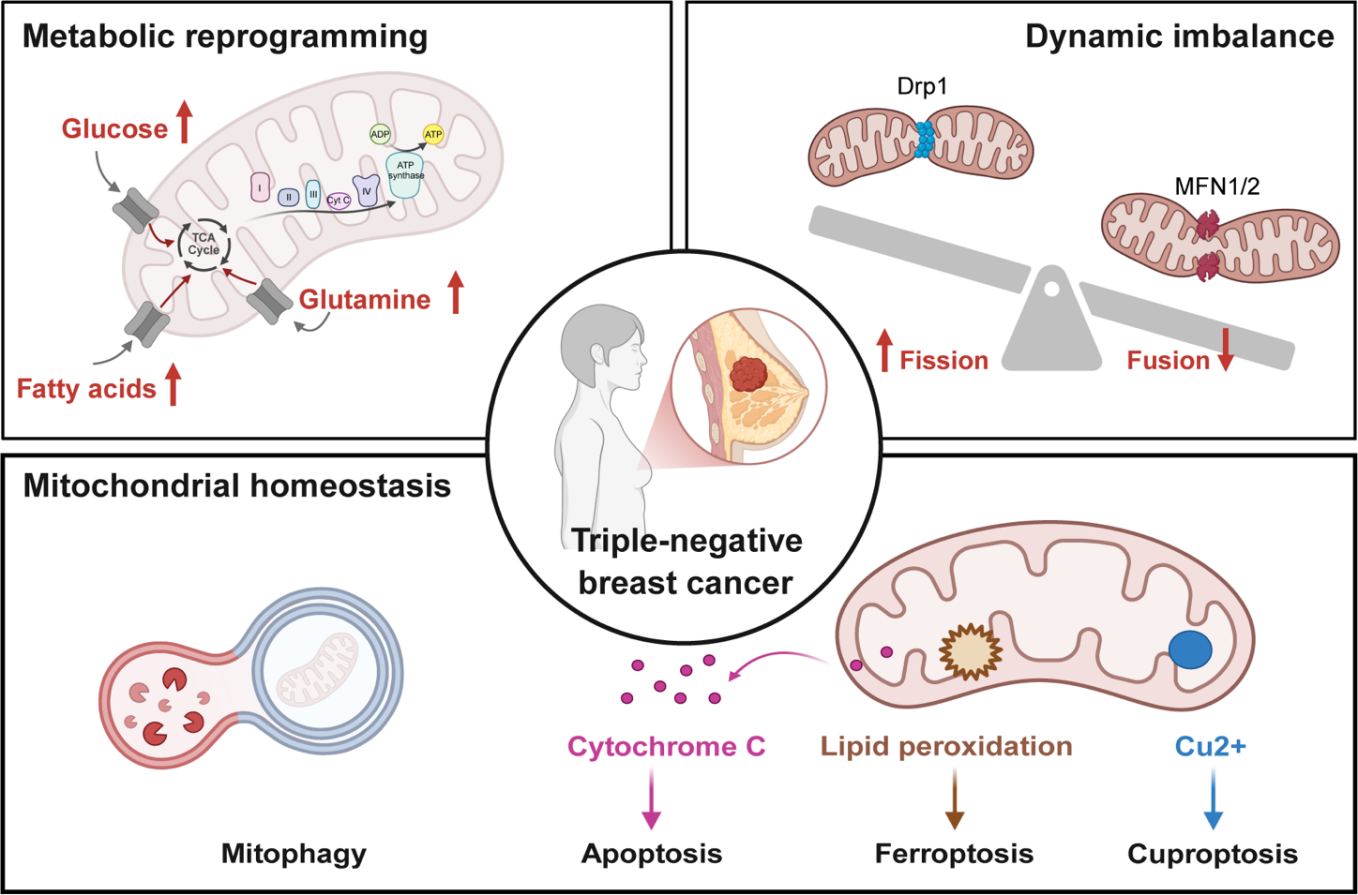
Schematic overview of the multifaceted roles of mitochondria in TNBC. Mitochondria serve as central hubs integrating multiple malignant processes in TNBC. Metabolic reprogramming involves the synergistic utilization of glucose, fatty acids, and glutamine to support tumor growth. Mitochondrial dynamics are regulated by fusion (MFN1/2) and fission (Drp1) proteins, whose imbalance contributes to metastasis and therapy resistance. Mitophagy acts as a double-edged sword, either promoting survival under stress or triggering cell death when overactivated. Mitochondria also orchestrate diverse programmed cell death pathways, including apoptosis, ferroptosis (driven by lipid peroxidation), and cuproptosis (induced by copper accumulation). This integrated network highlights mitochondria as a central vulnerability and therapeutic frontier in TNBC. (Figure was created with BioRender.com).

Mitochondria drive TNBC malignancy through an interconnected network of biological processes. As shown in **Figure 1**, these mechanisms include metabolic reprogramming—defined by the coordinated utilization of glucose, fatty acids, and glutamine to fuel the TCA cycle—along with dysregulated mitochondrial dynamics (fusion/fission), context-dependent mitophagy, and the orchestration of diverse programmed cell death pathways such as apoptosis, ferroptosis, and cuproptosis. This section systematically dissects these mitochondrial-driven mechanisms and their contributions to TNBC progression, metastasis, and therapeutic resistance.

#### 3.2.1 Synergistic orchestration of metabolic reprogramming

Metabolic reprogramming is a fundamental hallmark of cancer, arising from dysregulated signaling cascades that fulfill the energetic and biosynthetic demands of rapid tumor expansion (14, 15).While the classical Warburg effect posited that cancer cells preferentially rely on aerobic glycolysis due to impaired mitochondrial function (16), an expanding body of evidence reveals that many tumors, including TNBC, exhibit profound metabolic plasticity, switching to a metabolic program dominated by oxidative phosphorylation (OXPHOS) (17–20).

Multiple mechanisms underpin OXPHOS hyperactivation in TNBC. For instance, the regulator of cell cycle (RGCC) directs Polo-like kinase 1 (PLK1) to phosphorylate AMPKα2, a signaling cascade that synergistically co-activates OXPHOS and fatty acid oxidation (FAO), ultimately driving TNBC lung metastasis (21). Crucially, metabolic reprogramming skewed towards enhanced OXPHOS is a defining signature of acquired chemoresistance. In TNBC, the synergistic interplay between the MYC oncogene and myeloid cell leukemia-1 (MCL1) sustains the activity of chemoresistant breast cancer stem cells by hyperactivating mitochondrial OXPHOS (22), and dormant TNBC cells surviving chemotherapy exhibit a high-OXPHOS signature while relying on BCL-XL for survival (23). Collectively, these findings establish OXPHOS as a central vulnerability in the TNBC metabolic network.

**Figure 2:**
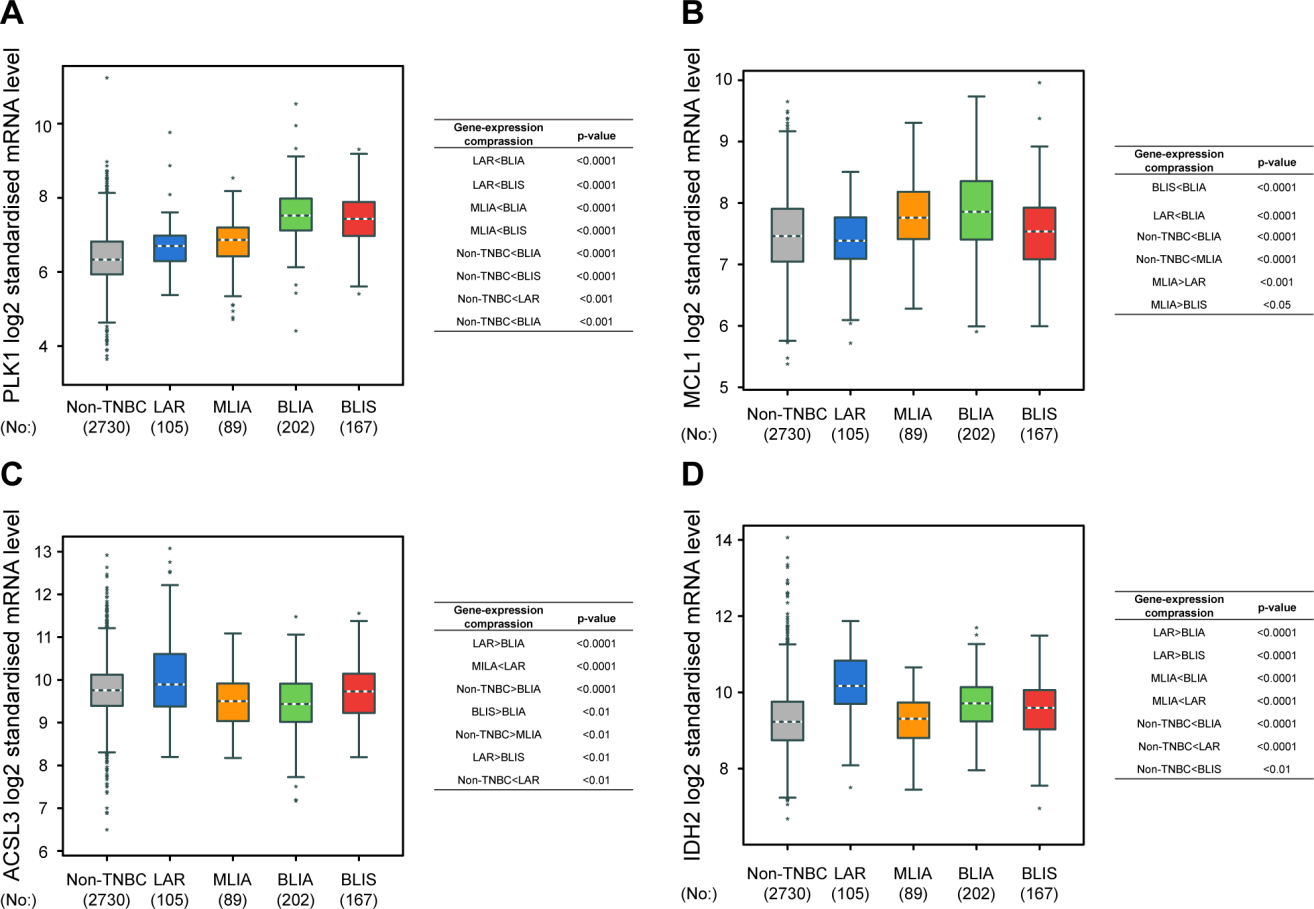
**(A)** PLK1 expression levels in non-TNBC and TNBC subtypes of clinical samples. **(B)** MCL1 expression levels in non-TNBC and TNBC subtypes of clinical samples. **(C)** ACSL3 expression levels in non-TNBC and TNBC subtypes of clinical samples. **(D)** IDH2 expression levels in non-TNBC and different subtypes of TNBC clinical samples. All data were derived from Affymetrix^®^, METABRIC database and analyzed using bc-GenExMiner v5.2. The cohorts include non-TNBC (n = 2730), LAR (n = 105), MLIA (n = 89), BLIA (n = 202), BLIS (n = 167). Statistical comparisons were performed using Welch’s test followed by Dunnett−Tukey−Kramer’s test where appropriate.

TNBC cells flexibly switch metabolic substrates under microenvironmental stress. Emerging evidence indicates that FAO is aberrantly hyperactivated in TNBC, driven by upregulation of fatty acid transporters and synthases. For example, long-chain acyl-coenzyme A synthetase 3 (ACSL3) scavenges oleic acid from mammary adipocytes to confer ferroptosis resistance (24). Beyond glucose and fatty acids, glutamine serves as an indispensable energy source, with TNBC exhibiting markedly higher glutamine dependency than other subtypes (25, 26). Isocitrate dehydrogenase 2 (IDH2) channels glutamine-derived α-ketoglutarate into the TCA cycle for lipid synthesis (27), and glutamine metabolism driving aggressive proliferation and invasion (28–30). To unravel the metabolic wiring underlying this heterogeneity, we interrogated the bc-GenExMiner v5.2 database. Intriguingly, we observed that the BLIA subtype exhibits significantly elevated expression of both PLK1 (**Figure 2A**), a kinase that co-activates OXPHOS and FAO, and MCL1 (**Figure 2B**), an anti-apoptotic protein that sustains OXPHOS in chemoresistant stem cells. This co-expression signature suggests that the BLIA subtype is particularly “addicted” to OXPHOS-driven metabolic programs, making it a prime candidate for therapeutic strategies aimed at disrupting mitochondrial respiration. Conversely, the LAR subtype displayed preferential upregulation of ACSL3 (**Figure 2C**) and IDH2 (**Figure 2D**). This distinct metabolic fingerprint indicates that the LAR subtype may rely more heavily on lipid and glutamine metabolism rather than simply glucose or OXPHOS.

Collectively, these data-driven observations underscore a critical concept: the internal molecular heterogeneity of TNBC directly dictates its metabolic preferences, implying that “one-size-fits-all” anti-mitochondrial strategies are likely to fail. Instead, subtype-specific metabolic vulnerabilities—such as OXPHOS addiction in BLIA versus ferroptosis susceptibility (via ACSL3) in LAR—must be therapeutically exploited.

#### 3.2.2 Dysregulation of mitochondrial dynamics

Mitochondria undergo continuous cycles of fusion and fission, processes indispensable for functional integrity (31). In TNBC, dysregulation of mitochondrial dynamics is intimately linked to metastasis, progression, and resistance (32, 33). For instance, silencing the fission regulator Dynamin-related protein 1 (Drp1, encoded by DNM1L) or overexpressing the Mitofusin 1 (MFN1) impairs lamellipodia formation and prevents mitochondrial recruitment to invasive fronts, thereby abrogating metastasis (34). Drp1 also establishes a positive feedback loop with Notch signaling, upregulating survivin and conferring apoptosis resistance (35).

However, the role of mitochondrial dynamics in TNBC is not unidirectional. Other evidence reveals a paradoxical picture: enhanced fission can suppress metastasis via ROS-dependent mechanisms (36), and metastatic TNBC cells often exhibit hyper-fused networks with upregulated glycolysis and lipid biosynthesis (37). We propose that three key factors explain these apparent contradictions. First, the experimental models employed—ranging from *in vitro* 2D cultures to xenografts—fail to recapitulate the complex condition of metastasis, where the energetic demands of dissemination, extravasation, and colonization differ drastically (38). Second, the functional outcome of Drp1 activity is exquisitely sensitive to its post-translational modifications (e.g., phosphorylation at Ser 616 vs. Ser 637), which are context-dependent and dictate whether fission promotes mitophagy (39). Third, the baseline metabolic heterogeneity of TNBC likely drives markedly differential responses to altered mitochondrial dynamics (40).

**Figure 3:**
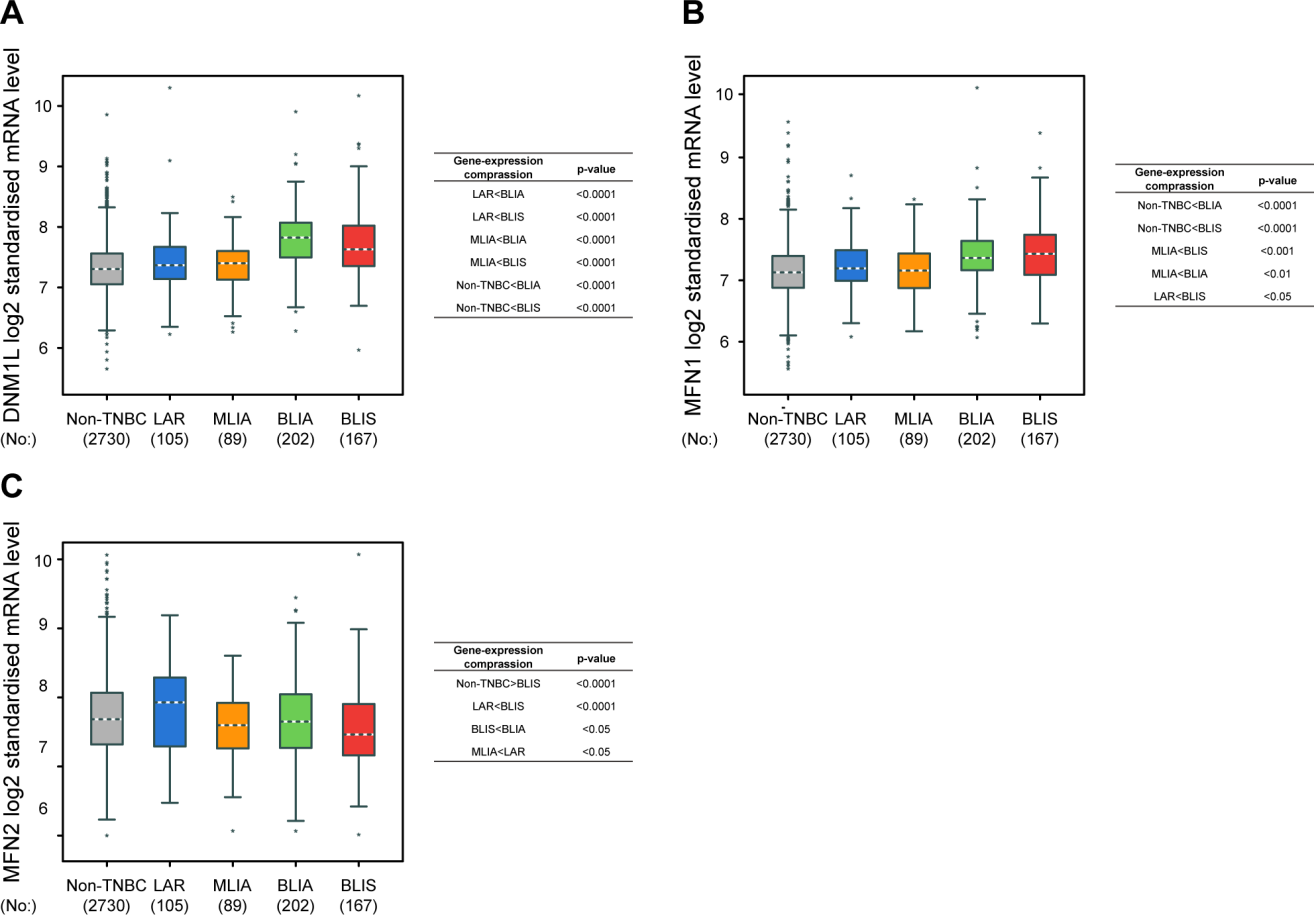
**(A)** DNM1L expression levels in non-TNBC and TNBC subtypes of clinical samples. **(B)** MFN1 expression levels in non-TNBC and TNBC subtypes of clinical samples. **(C)** MFN2 expression levels in non-TNBC and TNBC subtypes of clinical samples. All data were derived from Affymetrix^®^, METABRIC database and analyzed using bc-GenExMiner v5.2. The cohorts include non-TNBC (n = 2730), LAR (n = 105), MLIA (n = 89), BLIA (n = 202), BLIS (n = 167). Statistical comparisons were performed using Welch’s test followed by Dunnett−Tukey−Kramer’s test where appropriate.

Our bioinformatic analysis revealed subtype-specific mitochondrial dynamics. The BLIA subtype exhibited the highest expression of the fission gene DRP1 (**Figure 3A**), indicating active fragmentation. In contrast, the fusion genes MFN1 and MFN2 (**Figures 3B, C**) showed no simple reciprocal pattern but varied across subtypes. This dissociation suggests that the “mitochondrial dynamic balance” is uniquely tuned in each TNBC subtype—a high DRP1/low MFN state in BLIA may favor a fragmented, motile phenotype for local invasion, while other subtypes may require a fused network for metastatic colonization. Integrating these observations, we hypothesize a “spatiotemporal dependency” model: a transient increase in fission facilitates local invasion and migration, while a sustained hyper-fused state is required for successful metastatic outgrowth in distant organs.

However, the role of mitochondrial dynamics in TNBC is not unidirectional. Nutrient deprivation induces elongation to maintain ATP production (41). In TNBC, mitochondrial fission regulator 2 (MTFR2) frequently correlates with a glycolytic phenotype, suggesting a metabolic reliance on fragmented mitochondrial networks (42). Moreover, pyrroline-5-carboxylate reductase 3 (PYCR3) augments mtDNA copy number and respiration, driving proliferation and doxorubicin resistance (43), while horizontal transfer of mutated mtDNA via extracellular vesicles disseminates chemoresistance (44). Collectively, these findings provide compelling evidence that mitochondrial dynamics function as a multifaceted regulator that dictates TNBC progression through an intricate interplay of morphology, metabolism, and genomic stability. Thus, deciphering these mitochondrial dynamic features will be essential for designing personalized therapies that either tip the balance toward excessive fission to trigger cell death, or stabilize fusion to block metastasis.

#### 3.2.3 Mitochondrial quality control: the double-edged sword of mitophagy

Mitochondrial Quality Control (MQC) is a fundamental homeostatic mechanism for preserving mitochondrial functional integrity. At its core, mitophagy—the selective sequestration and degradation of damaged or dysfunctional mitochondria—acts as a primary checkpoint for mitochondrial fidelity (9). In TNBC, the role of mitophagy is increasingly recognized as dichotomous and highly context-dependent, serving as both an adaptive survival mechanism and a potential lethal trigger (45).

Under microenvironmental stress, moderate mitophagy promotes survival. For instance, hypoxic tumor microenvironment leads to mitochondrial accumulation of pyruvate dehydrogenase kinase 1 (PDK1), driving chemoresistance by hyperactivating mitophagy (46). In stark contrast, mitophagy defects resulting from BNip3 loss accelerate metastasis (47), revealing that mitophagy exerts opposing, context-dependent functions in TNBC. In addition, excessive mitophagy can precipitate cell death; robust expression of UCP1 suppresses TNBC progression by activating both mitophagy and pyroptosis (48). Consequently, fine-tuning the intensity of mitophagy—shifting it from a survival-oriented response to a pro-death signal—represents a sophisticated strategy for targeted therapy. Notably, the activation of mitophagy is frequently coupled with profound metabolic shifts. A deeper exploration of the reciprocal crosstalk between metabolic reprogramming and mitophagy will be imperative for fully deciphering the core regulatory logic of MQC and for developing more potent therapeutic modalities.

#### 3.2.4 Mitochondria execute diverse programmed cell death pathways

Classically, mitochondria are recognized as the primary executioners of intrinsic apoptosis. However, the recent discovery of novel programmed cell death (PCD) modalities—including ferroptosis, cuproptosis, and necroptosis—has unveiled mitochondria as central signaling hubs orchestrating these diverse pathways (49–51). Dysregulation of these PCD mechanisms is intimately associated with the malignant progression and therapeutic recalcitrance of TNBC.

In TNBC, apoptotic evasion is critical. Overexpression of anti-apoptotic proteins (Bcl-2, Bcl-xL) neutralizes pro-apoptotic mediators and blunts cytochrome c release, driving chemoresistance (52, 53).

**Figure 4:**
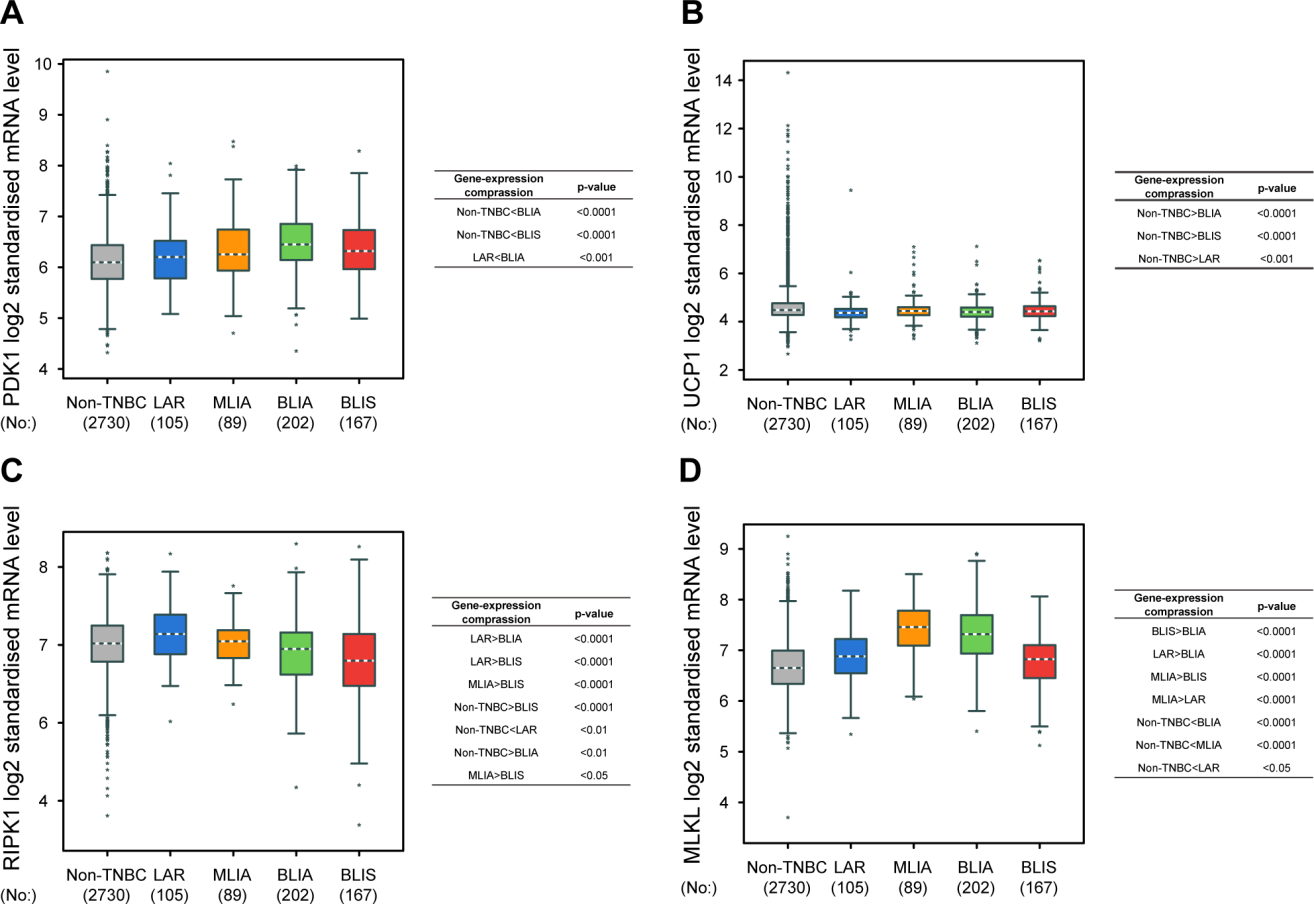
**(A)** PDK1 expression levels in non-TNBC and TNBC subtypes of clinical samples. **(B)** UCP1 expression levels in non-TNBC and TNBC subtypes of clinical samples. **(C)** RIPK1 expression levels in non-TNBC and TNBC subtypes of clinical samples. **(D)** MLKL expression levels in non-TNBC and different subtypes of TNBC clinical samples. All data were derived from Affymetrix^®^, METABRIC database and analyzed using bc-GenExMiner v5.2. The cohorts include non-TNBC (n = 2730), LAR (n = 105), MLIA (n = 89), BLIA (n = 202), BLIS (n = 167). Statistical comparisons were performed using Welch’s test followed by Dunnett−Tukey−Kramer’s test where appropriate.

Ferroptosis, driven by lipid peroxidation following GPX4 inactivation (54), exhibits high heterogeneity across TNBC. The luminal androgen receptor (LAR) subtype, characterized by the upregulation of oxidized phosphatidylethanolamines and glutathione metabolism (especially GPX4), allows the utilization of GPX4 inhibitors to induce ferroptosis (55). Cuproptosis is triggered by copper binding to lipoylated TCA cycle components, with mitochondria occupying a focal position given TCA enzyme compartmentalization (56–58). Necroptosis, mediated by receptor-interacting serine/threonine kinases 1 and 3 (RIPK1, RIPK3) and mixed lineage kinase domain-like protein (MLKL) (49), which can promote vasculogenic mimicry in TNBC (59). Taken together, as the central regulatory platform for multiple PCD modalities, mitochondria deeply dictate the malignant trajectory and chemoresistance of TNBC. Given this complexity, we wonder whether these pathways are uniformly wired across TNBC subtypes. However, our bioinformatic analysis reveals that not all TNBC subtypes share the same mitochondrial death circuit (**Figure 4**). PDK1 is highest in BLIA (**Figure 4A**), suggesting that BLIA cells rely on PDK1-driven mitophagy to survive. UCP1 peaks in non-TNBC (**Figure 4B**), meaning this pyroptosis-inducing pathway is largely lost in TNBC. For necroptosis, RIPK1 is highest in LAR (**Figure 4C**) while MLKL peaks in MLIA (**Figure 4D**)—a clear functional decoupling. In short, mitochondrial-dependent cell death is wired differently across subtypes.

Therefore, we propose subtype-guided approaches: inhibit PDK1 in BLIA to block protective mitophagy; restore UCP1 to trigger pyroptosis; and in LAR or MLIA tumors, induce necroptosis via RIPK1/MLKL. These tailored interventions may bypass the universal apoptotic resistance of TNBC more effectively than blanket therapies.

### 3.3 Targeting mitochondria: from mechanistic insights to translational therapeutic strategies

Given the central role of mitochondria in the malignant progression of TNBC, the development of mitochondria-targeted therapeutic strategies has emerged as a frontline research priority. These approaches encompass a broad spectrum of interventions, ranging from the direct induction of mitochondrial dysfunction to the sophisticated indirect modulation of mitochondrial metabolism and dynamics (**Table 1**).

**Table 1:** Summary of mitochondria-targeted therapeutic strategies in TNBC.

Therapeutic strategy	Mitochondrial target/mechanism	Representative agents/interventions	Biological effects	References
	Mitochondrial Complex I	Coptisine	Inhibits Complex I activity, reduces ATP production	(61)
OXPHOS inhibition	Mitochondrial Complex III	DHODH inhibitors	Induces S-phase arrest and mitochondrial dysfunction in complex III	(60)
	Mitochondrial protease ClpP	TR-107	Hyperactivates ClpP, degrades mitochondrial proteins	(64)
Glutamine metabolism targeting	Glutaminase inhibitor + FAO inhibitor	Glutaminase inhibitors + CPT1 inhibitors	Sensitizes glutaminase inhibitor-resistant TNBC cells	(67)
	Drp1	Mdivi-1, Reniformin A	Inhibits mitochondrial fission	(68, 69)
Mitochondrial dynamics modulation	FIS1	Depletes tumor-initiating cell population	Depletes tumor-initiating cell population	(70)
	PGC-1α	Shikonin	Promotes PGC-1α degradation, suppresses CAF-induced metastasis	(73)
Mitophagy regulation	Mitochondrial damage	Chloroquine	Induces mitochondrial damage, impairs DNA repair in cancer stem cells	(77)

Abbreviations: OXPHOS, oxidative phosphorylation; ClpP, caseinolytic protease P; DHODH, dihydroorotate dehydrogenase; FAO, fatty acid oxidation; CPT1, carnitine palmitoyltransferase 1; Drp1, dynamin-related protein 1; FIS1, mitochondrial fission 1 protein; PGC-1α, peroxisome proliferator-activated receptor gamma co-activator 1-alpha; CAF, cancer-associated fibroblast.

#### 3.3.1 Disrupting bioenergetic and biosynthetic sources: targeting core metabolic pathways

Direct pharmacological intervention in OXPHOS represents a promising therapeutic avenue. A diverse array of compounds targeting Complex I, Complex III, or mitochondrial proteostasis exerts anti-tumor effects in TNBC models (60–64). Crucially, OXPHOS inhibitors have proven effective in overcoming chemoresistance in TNBC, underscoring the clinical feasibility of targeting mitochondrial respiration (65). However, the profound metabolic plasticity of TNBC presents a formidable challenge to monotherapy. While an OXPHOS inhibitor may initially suppress tumor growth, the selective pressure it exerts inevitably drives the emergence of resistant clones that have re-wired their metabolism to rely on glycolysis or alternative fuel sources. To overcome this, we must move beyond the one-size-fits-all approach. Future clinical trials should be designed to prospectively stratify patients based on their tumor’s metabolic profile, as determined by pre-treatment metabolomics or functional imaging. In light of the profound metabolic plasticity exhibited by TNBC, the development of bifunctional molecules that simultaneously target glycolysis and mitochondrial function may yield superior synergistic efficacy. A notable example includes bifunctional metallic probes designed to inhibit glycolysis (e.g., via lonidamine) while concurrently disrupting mitochondrial function by targeting the interaction between hexokinase 2 (HK2) and the voltage-dependent anion channel 1 (VDAC1) (66). Additionally, the dual inhibition of glutaminase and CPT1 has been shown to sensitize glutaminase inhibitor-resistant TNBC cells (67). Collectively, targeting the specific metabolic dependencies of TNBC—particularly key mitochondrial metabolic nodes—offers a transformative strategic direction for reversing drug resistance and pioneering the next generation of cancer therapeutics.

#### 3.3.2 Remodeling mitochondrial dynamic homeostasis

While mitochondrial dynamics are inherently characterized by continuous and fluid transitions, mounting evidence suggests that pharmacological inhibition of mitochondrial fission exerts robust anti-tumor effects in TNBC. For instance, Reniformin A has been shown to suppress TNBC progression by inducing DRP1-mediated mitochondrial dysfunction and subsequent apoptosis (68). Similarly, the Drp1 inhibitor Mdivi-1 significantly potentiates the cytotoxic efficacy of paclitaxel (69), while targeted intervention against FIS1, a critical mediator of mitochondrial fission, effectively depletes the pool of tumor-initiating cells (70). Notably, therapeutic strategies targeting fission frequently converge with metabolic suppression; BET inhibitors alter OXPHOS by modulating mitochondrial dynamics (71), and cepharanthine sensitizes TNBC to epirubicin by inducing fission-mediated apoptosis (72). In addition, mitochondrial biogenesis also serves as a pivotal regulatory node within the landscape of mitochondrial dynamics. Research indicates that shikonin can intercept metastasis induced by cancer-associated fibroblasts (CAFs) in TNBC by mediating the phosphorylation-dependent degradation of peroxisome proliferator-activated receptor-gamma co-activator-1alpha (PGC-1α) (73). Thus, remodeling mitochondrial dynamic homeostasis—particularly through the strategic inhibition of mitochondrial fission—has emerged as a compelling innovative strategy to suppress TNBC metastasis and overcome therapeutic resistance.

#### 3.3.3 Synergistically activating multiple cell death modalities

Precision modulation of mitophagy is paramount for therapeutic success, as inhibiting cytoprotective mitophagy has been shown to restore chemosensitivity. For instance, the combination of miR-218-5p and doxorubicin enhances anti-cancer activity by suppressing Parkin-dependent mitophagy (74). Meanwhile, blockade  pro‑survival mitophagy can directly precipitate cell death; for example, sonodynamic therapy suppresses TNBC progression by eliminating mitophagy flux (75). Furthermore, novel synthetic small molecules and delivery systems offer innovative avenues for activating therapeutic mitophagy. A redox-responsive dendritic copolymer-drug conjugates has been engineered to enhance therapeutic mitophagy by synergizing with microtubule destabilization (76). Autophagy inhibitors such as chloroquine target TNBC stem cells by inducing mitochondrial damage and impairing DNA break repair (77). In combinatorial settings, Pimavanserin tartrate has been shown to induce both apoptosis and cytoprotective autophagy (78). Moreover, given the critical role of endoplasmic reticulum (ER) stress in inducing TNBC apoptosis, “organelle crosstalk” therapies—simultaneously targeting the ER-mitochondria axis—have proven effective in treating multidrug-resistant breast cancer (79).

Simultaneously, extensive research is dedicated to developing compounds capable of reactivating the mitochondrial apoptotic pathway. Natural products such as epoxyazadiradione (80), and bruceine A (81) trigger apoptosis by inducing the dissipation of mitochondrial membrane potential and the subsequent release of cytochrome c. Synthetic small molecules like Disarib (a selective BCL2 inhibitor) (82) and various metal complexes have also exhibited potent pro-apoptotic activity (83–85). Additionally, “drug repurposing” strategies have identified antipsychotics—such as trifluoperazine (86) and fluphenazine (87)—as potent inhibitors of TNBC growth via the mitochondrial apoptotic pathway, providing a diverse toolkit to overcome apoptotic resistance.

Currently, multiple strategies are being deployed to induce diverse mitochondria-dependent cell death modalities, yielding promising anti-tumor outcomes in TNBC models. For example, sulfasalazine effectively triggers autophagy-related ferroptosis (88). while bioactive components from traditional Chinese medicine, such as α-hederin (89), and resveratrol (90) have been shown to induce ferroptosis by modulating GPX4 or iron metabolism. Strategies centered on cuproptosis involve either leveraging copper ionophores to increase intracellular copper accumulation (57), or combining copper overload with the amplification of oxidative stress (91, 92).

Given the inherent heterogeneity and plasticity of tumors, a single-modality death induction strategy may be insufficient. Therefore, the simultaneous induction of apoptosis, ferroptosis, and pyroptosis may yield powerful synergistic effects. For instance, copper-iron bimetallic nanosheets can concurrently trigger cuproptosis and ferroptosis (93), while arsene-vanadene nanodots enhance chemo-immunotherapy by co-activating apoptosis and ferroptosis (94). This “multifaceted” approach holds significant potential to further dismantle the chemoresistance of TNBC.

### 3.4 Mitochondrial-related biomarkers: new horizons in TNBC stratification and therapeutic monitoring

Accumulating evidence suggests that mitochondrial genetic and functional aberrations endow aggressive TNBC with distinct molecular signatures (95). Consequently, identifying mitochondrial-related biomarkers is of paramount importance for the stratification of TNBC patients and the real-time monitoring of therapeutic efficacy. Specifically, prognostic models—or “signatures”—constructed based on mitophagy-related genes have been validated as effective tools for predicting both the survival outcomes and the immunotherapy responses of TNBC patients (96, 97). Moreover, metastatic TNBC cells are closely associated with a fused (elongated and interconnected) mitochondrial morphology, whereas non−metastatic TNBC cells tend to exhibit a more fragmented mitochondrial morphology (37). This distinct association not only underscores the pivotal role of mitochondrial dynamics in TNBC progression but also highlights the potential of these regulatory nodes to serve as novel therapeutic targets (32).

On the technological frontier, the integration of dynamic light scattering (DLS) microscopy with deep learning algorithms has enabled the label-free detection of mitochondrial dynamics within TNBC cells (98). Concurrently, investigations into the impact of platelet-derived mitochondrial transfer on the metabolic profiling and aggressive progression of metastatic TNBC cells have further expanded our understanding of the mitochondrial regulatory network (99). In terms of clinical imaging, 7T multi-parametric magnetic resonance imaging (MRI) has been employed to monitor the therapeutic efficacy of inositol in teprenone-resistant TNBC mouse models. This advanced imaging approach has facilitated a deeper exploration of how inositol overcomes teprenone resistance and its intrinsic link to remodeling mitochondrial morphology (100). Collectively, these advancements demonstrate that a profound analysis of mitochondrial function and dynamics not only refines the molecular subtyping and therapeutic assessment of TNBC but also offers transformative perspectives for developing personalized, mitochondria-targeted treatment strategies.

### 3.5 Conclusion and perspectives

Mitochondria have evolved far beyond their traditional identity as mere “cellular powerhouses,” emerging as the central regulatory hubs driving the malignant progression, metastasis, stemness maintenance, immune evasion, and therapeutic resistance of TNBC. The intricate functional landscape of mitochondria—encompassing metabolic plasticity, dynamic remodeling, quality control, mtDNA homeostasis, ROS signaling, and inter-organelle communication networks—constitutes the biological bedrock of treatment resistance in TNBC. Consequently, targeting mitochondria represents a transformative frontier in TNBC therapy. A multitude of diversified strategies—ranging from the direct inhibition of core metabolic pathways like OXPHOS and FAO to the induction of specific cell death modalities (apoptosis, ferroptosis, and cuproptosis) via small molecules or nanomaterials, and from the fine-tuning of mitochondrial dynamics to the deployment of advanced precision delivery systems—has demonstrated immense potential in preclinical settings. Furthermore, lifestyle interventions, such as short-term fasting (101), moderate exercise (102), and dietary folate restriction (103) have also emerged as promising adjunctive approaches.

Despite these advancements, significant challenges remain on the path to clinical translation. First and foremost, given the indispensable role of mitochondria in physiological cellular functions, achieving a sufficient therapeutic window to selectively eradicate TNBC cells while sparing healthy tissue—thereby minimizing “off-target” toxicities—is a critical hurdle. Secondly, the profound heterogeneity and metabolic plasticity inherent to TNBC suggest that monotherapy targeting a single mitochondrial node may rapidly lead to adaptive resistance. Future studies should more thoroughly characterize the mitochondrial signatures that distinguish different TNBC subtypes. (95, 104, 105).

This will also facilitate the development of biomarker-guided, personalized combination regimens, such as the synergistic integration of mitochondria-targeted agents with immunotherapy (106), chemotherapy (107), targeted therapy (108) or even the co-administration of distinct mitochondrial inhibitors (109). Consequently, We urgently need to develop and clinically validate a comprehensive “mito-signature” panel—integrating tumor metabolic profiling (e.g., OXPHOS vs. FAO dependency via mass spectrometry imaging), mitochondrial DNA mutation burden, and live-cell imaging of mitochondrial morphology from patient biopsies. Such a panel would enable the prospective identification of TNBC patients most likely to benefit from specific mitochondria-targeted regimens, forming the foundation for adaptive, biomarker-driven clinical trials.

In addition to targeting the organelle directly, we must explore the broader ecosystem. Recent evidence shows that platelet-derived mitochondria can be transferred to TNBC cells, altering their metabolic fitness (99). Can we therapeutically block this “mitochondrial donation” from stromal cells or platelets to tumor cells, thereby sensitizing them to metabolic stress? The horizontal transfer of mutated mtDNA via extracellular vesicles confers chemoresistance (44). We need to investigate whether the mtDNA variant load within a tumor can serve as a predictive biomarker for response to OXPHOS inhibitors and whether we can intervene to block this non-genetic form of resistance. Furthermore, it is now clear that mitochondrial fission and fusion dictate the metabolic fitness and anti-tumor function of T-cells and macrophages (110–113). A key question is whether we can selectively modulate mitochondrial dynamics in immune cells to enhance their tumoricidal activity, without negatively impacting the immune cells themselves.

In conclusion, as our understanding of mitochondrial biology continues to deepen, targeting this organelle is poised to become an indispensable pillar of the future TNBC treatment landscape. By embracing a strategy centered on dynamic biomarker-guided patient selection, rational combinatorial regimens, and exploration of the broader mitochondrial ecosystem, we can translate these mechanistic insights into effective and durable clinical outcomes for patients.

## Data Availability

The original contributions presented in the study are included in the article/supplementary material. Further inquiries can be directed to the corresponding author/s.
